# Editorial: Cardiovascular calcification: disease mechanisms, clinical phenotypes and therapeutic strategies

**DOI:** 10.3389/fcvm.2026.1820272

**Published:** 2026-03-27

**Authors:** Lukas Nollet, Georges Leftheriotis, Olivier M. Vanakker

**Affiliations:** 1 Center for Medical Genetics Ghent, Ghent University Hospital, Ghent, Belgium; 2 Department of Biomolecular Medicine, Ghent University, Ghent, Belgium; 3 Vascular Physiology and Medicine Unit – Rare Arterial Diseases Center, University Hospital of Nice, Nice, France

**Keywords:** atherosclerosis, biomarker, calcific aortic valve disease (CAVD), calcification, pathophysiology, treatment

Cardiovascular disease is the leading cause of morbidity and mortality worldwide and is associated with significant healthcare costs (1). Despite major advances in the fields of preventive cardiology, coronary interventions and heart failure management, the residual burden of cardiovascular disease remains high.

Cardiovascular calcification (CVC) presents a significant contributor to this residual disease risk as shown by numerous studies linking CVC to adverse cardiovascular outcomes, independent of traditional risk factors such as arterial hypertension, dyslipidemia and obesity (2, 3).

Once considered a benign epiphenomenon of cardiovascular ageing, CVC is now widely regarded as a pathological process driving vascular stiffness, atherosclerotic plaque instability, and valvular dysfunction. Indeed, the abnormal deposition of calcium phosphate crystals, mainly hydroxyapatite, in the vascular wall, cardiac valves or myocardium inflicts a vicious cycle of local tissue damage which further enhances the calcification process (4).

Key mechanisms contributing to CVC include osteochondrogenic differentiation of mesenchymal cells, reduced synthesis or accelerated breakdown of anti-calcifying factors such as inorganic pyrophosphate (PPi) and matrix-gla protein (MGP), and increased production of reactive oxygen species (ROS) and pro-calcifying extracellular vesicles (5).

The global rise in diabetes mellitus and chronic kidney disease (CKD), which are both invariably linked to CVC, has prompted an increased awareness of the debilitating effects of CVC on cardiovascular health in these high-risk populations, yet effective therapies preventing or reversing CVC are still lacking.

Rare monogenic CVC disorders such as pseudoxanthoma elasticum (PXE) and generalized arterial calcification of infancy (GACI) present excellent models to study the complex pathophysiology of CVC both *in vitro* and *in vivo* (6).

In this Research Topic, fourteen original and review articles are presented, spanning the entire and diverse field of CVC research including basic, translational and clinical studies.

## Basic research on vascular calcification

Highlighting the importance of extracellular vesicles in CVC pathogenesis, Li et al. revealed that bone marrow mesenchymal stem cell-derived exosomes containing microRNA-335 attenuate CVC by inhibiting SP1 and RUNX2 expression. AgomiR-335 treatment in a rat model significantly reduced aortic calcification burden. This study shows that multi-organ mechanisms are involved in CVC pathophysiology, demonstrating an important cross-talk between the bone marrow and arterial blood vessels.

Excessive inflammatory responses drive the formation of atherosclerotic plaques and subsequent calcification. Interferon regulartory factors (IRFs) play a crucial role in modulating these inflammatory mechanisms, as described in the review article of Wang et al. IRF8 in particular has been shown to regulate CVC by stimulating the transdifferentiation of vascular smooth muscle cells towards macrophages, promoting matrix degradation through MMP9, forming a nidus on which calcium crystal deposition can occur. Indeed, the pathogenic role of MMP9 in CVC is widely recognized, as indicated by earlier studies in the Mendelian disorder PXE (7, 8).

Similar to the interferon family of cytokines, interleukins have been shown to be an integral part of immune signaling-related pathological mineralization as described by Zhao et al. In a clinical context, interleukins can be easily targeted by monoclonal antibodies such as ziltivekimab, an IL-6-inhibiting protein whose efficacy in reducing cardiovascular disease is currently under investigation in the multinational placebo-controlled ZEUS trial (NCT05021835) including patients with established atherosclerotic cardiovascular disease, CKD and systemic inflammation (9).

Extracellular matrix components, in particular elastic fibers, play a fundamental role in CVC pathogenesis by forming a scaffold on which calcium crystal formation can be initiated. In their comprehensive review article, Lofaro et al. provide an overview on elastic fiber homeostasis and disruption in CVC and highlight the importance of elastic fiber-associated molecules such as fibrillins and proteoglycans.

Interestingly, treatment with nanoparticles containing calcium chelating agents and which are coated with antibodies specifically binding to degraded elastin has shown promising results in CVC models (10). These nanoparticles carry potential for becoming the first therapy which can effectively resolve already existing hydroxyapatite crystal deposits, partially restoring the physiological biomechanical and molecular signaling properties of elastic fibers.

## Clinical research on vascular calcification

At the clinical level, the work from Deng and Qin shed further light on the association between obesity and CVC (Deng and Qin). The authors found an inverse U-shaped association between lipid accumulation product index and abdominal aortic calcification in a national cohort of nearly 3,000 participants. While hormonal disturbances, including altered leptin levels, have been shown to contribute to CVC pathogenesis by increasing osteochondrogenic transdifferentiation of vascular smooth muscle cells and activating RUNX2, it remains unknown why a very high lipid accumulation product index seems to protect individuals from CVC (11).

Similarly, Tan et al. showed that the Naples prognostic score, comprising serum albumin and total cholesterol levels as well as leukocyte ratios, significantly and positively associated with severe abdominal aortic calcification. These leukocyte ratios are of particular interest, as excessive inflammation is a known risk factor for CVC development. Additionally, impaired resolution of the inflammation response has also been identified in calcific aortic valve disease (CAVD) and atherosclerosis (12).

## Basic research on CAVD

CAVD is a major cause of structural heart disease and identifying its genetic architecture may improve understanding and treatment. Using bulk RNA sequencing, Wang et al. identified 16 hub genes, including ITGB3, ITGAV, and MMP9 and demonstrated good diagnostic performance for CAVD. Furthermore, MMP9 and PLAU likely contribute to disease progression by promoting inflammatory responses in monocytes and macrophages.

Preclinical models play a pivotal role to study CAVD. In their extensive review Lafosse et al. assessed their strengths and limitations and how they can be used to investigate the cellular and molecular mechanisms involved in CAVD.

The central role of macrophages in CAVD was reviewed by Issa et al., highlighting the opposing effects of inflammatory macrophages—which promote the osteogenic transformation of valvular interstitial cells (VIC) though the release of numerous cytokines—and immunomodulatory macrophages, which release PPi hence protecting against valvular calcification.

The potential of nutrient restriction (NR) to mitigate VIC calcification through the ubiquitin-proteasome system (UPS) was studied by Phadwal and co-authours (Phadwal et al.). NR significantly downregulated osteogenic markers and decreased calcium deposition in rat VICs. This study highlights NR as a promising non-invasive intervention for preventing VIC calcification by modulating the UPS.

## Novel therapies for CVC

As highlighted by O’Brien et al., medial vascular calcification remains a major unmet therapeutic challenge, particularly in genetic disorders, diabetes mellitus, and CKD. Central to emerging strategies is restoration of PPi homeostasis and approaches ranging from direct PPi supplementation and bisphosphonates to recombinant ENPP1 replacement and alkaline phosphatase inhibition illustrate a shift toward mechanism-based therapies aimed at correcting metabolic drivers of calcification. Adjunctive strategies including phosphate binders, magnesium, and vitamin K further emphasize the multifactorial nature of mineral imbalance in ectopic calcification.

Beyond prevention, advances in interventional cardiology are reshaping management of established calcified lesions. Zhang et al. demonstrate that intravascular lithotripsy may preserve coronary microvascular function more effectively than rotational atherectomy, as reflected by lower post-procedural microvascular resistance following PCI in heavily calcified vessels. Although clinical event differences were not statistically significant, these findings suggest that plaque-modifying technologies can influence downstream microvascular biology, highlighting the importance of procedural strategies that minimize embolization and inflammatory injury associated with calcium disruption.

In a broader context of vascular health, the study by Feng et al. revealed that aging and prolonged cold exposure synergistically impair vascular healing after drug-eluting stent implantation in elderly patients, leading to delayed endothelialization and excessive neointimal growth, ultimately increasing the risk of major adverse cardiovascular events. Using serial optical coherence tomography imaging and environmental data, the authors propose a novel 4D risk score for personalized risk stratification.

Finally, Bergeon et al. expand the therapeutic scope by linking CAVD with inflammation and autonomic dysregulation, proposing preoperative transcutaneous vagus nerve stimulation as a novel preventive strategy for postoperative atrial fibrillation. By enhancing parasympathetic tone and promoting resolution of inflammation, neuromodulation may address both arrhythmogenic substrates and inflammatory pathways associated with CAVD.

## Advancing CVC research through global collaboration

To conclude, we would like to highlight the importance of multinational interdisciplinary research collaborations in the field of CVC, which can greatly accelerate research progress.

Both the International Network on Ectopic Calcification (INTEC, http://www.itnintec.com) and the International Scientific Society of Ectopic Calcification (ISSEC, http://www.issec.org) have been created to this end and comprise both basic and clinical researchers as well as patient advocacy groups and representatives from the pharmaceutical industry. Scientific work of multiple INTEC/ISSEC members has been included in this Research Topic.

These networks may significantly advance fundamental and clinical research discoveries and their translation towards the ultimate objective: a safe, affordable, and effective treatment for patients with CVC disorders.
